# A Case of Abortion, and Retention of the Placenta

**Published:** 1846-01

**Authors:** A. R. Nelson

**Affiliations:** Rutherford county, Tennessee


Art. III.—*A Case of Abortion, and Retention of the Placenta.* By A. R. Nelson, M.D., of Rutherford county, Tennessee.

On the night of the 17th July last, I was called to see a negro woman supposed to be in labor. On arriving, I found that the fœtus had already been expelled. From its appearance and size, I supposed it to be from four and a half to five months advanced.

After waiting the ordinary length of time and finding that no efforts were made on the part of the uterus for the expulsion of its contents, the usual measures of an external and tangible character were resorted to, for the accomplishment of this object, but all to no effect.

Seeing the insufficiency of these measures, remedies of an internal character were next administered, and as a matter of course a dose of the secale cornutum was given, but the article produced no effect. On the morning of the 18th a second dose, consisting of half a drachm, was administered, without producing the slightest effect. During the afternoon blood was drawn to the amount of ten or twelve ounces; a dose of oil was given which operated well. The external organs of generation were very much swollen as was also the abdomen.

She informed me that a day or two previous to her confinement, œdema of the lower extremities had existed. Warm fomentations were applied to the parts, and continued through the day. Her pulse was full and frequent from the commencement, but was reduced by the abstraction of blood.

Friction was applied from the first to the last, but no contraction followed; twenty-four hours after the expulsion of the fœtus, the hook was resorted to, and by means of it a portion of the membranes brought away; during the day the umbilical cord detached itself. At late bed-time ten grains of blue mass, with half a grain of ipecacuanha were administered.

On the morning of the 19th I visited her, and was informed that she had rested pretty well the previous night. Her pulse was rather frequent than otherwise. During the night she felt some sickness at the stomach, which, I suppose, was caused by the medicine given. Between midnight and day she had an alvine evacuation; an hour by sun this was followed by a second, but not understanding that her bowels had been sufficiently moved, a dose of salts was ordered. This produced, however, only two passages. About eleven o'clock in the morning she was reported to have had a chill; after which she became very restless and remained so during the day, complaining of headache which was attended by slight fever.

At night examination per vaginam was made, and the parts were found to be still in a tumid condition. The common hook was again resorted to, and with no little difficulty the placenta, together with the remaining portion of membranes was extracted, in an hour or two afterwards. The patient soon became more quiet, and under the influence of five grains of Dover's powder and ten of blue mass, was left for the night.

On the morning of the 20th I found her comfortable, and learned that she had rested well during the night. Her pulse was in a better condition, and her situation was in every respect greatly improved.

I will merely add, that this woman was thirty-seven years of age, and that this was her ninth pregnancy, and seventh abortion. Her health appears to be good, except in this respect.

November, 1845.

